# The Role of Prospection in Steep Temporal Reward Discounting in Gambling Addiction

**DOI:** 10.3389/fpsyt.2015.00112

**Published:** 2015-08-25

**Authors:** Antonius Wiehler, Uli Bromberg, Jan Peters

**Affiliations:** ^1^Department of Systems Neuroscience, University Medical Center Hamburg-Eppendorf, Hamburg, Germany

**Keywords:** addiction, pathological gambling, temporal discounting, delay discounting, autobiographical memory, episodic future thinking, time perception

## Abstract

Addiction and pathological gambling (PG) have been consistently associated with high impulsivity and a steep devaluation of delayed rewards, a process that is known as temporal discounting (TD). Recent studies indicated that enhanced episodic future thinking (EFT) results in less impulsive TD in healthy controls (HCs). In a separate line of research, it has been suggested that non-linearities in time perception might contribute to reward devaluation during inter-temporal choice. Therefore, in addition to deficits in valuation processes and executive control, impairments in EFT and non-linearities in time perception have been hypothesized to contribute to steep TD in addiction. In this study, we explore such a potential association of impairments in EFT and time perception with steep TD in PG. We investigated 20 PGs and 20 matched HCs. TD was assessed via a standard computerized binary choice task. EFT was measured using a variation of the Autobiographical Memory Interview by Levine et al. (1). Time perception was assessed with a novel task, utilizing a non-linear rating procedure via circle-size adjustments. Groups did not differ in baseline EFT. In both groups, a power law accounted time perception best, and the degree of non-linearity in time perception correlated with discounting across groups. A multiple regression analysis across all predictors and covariates revealed that only group status (PG/HC) and depression were significantly associated with discounting behavior such that PG increased TD and depression attenuated TD. Our findings speak against the idea that steep TD in PG is due to a skewed perception of time or impairments in EFT, at least under the present task conditions. The lack of overall group differences in EFT does not rule out the possibility of more complex interactions of EFT and decision-making. These interactions might be diminished in pathological gambling or addiction more generally, when other task configurations are used.

## Introduction

Throughout life, we are faced with numerous choices between tempting immediate rewards and larger rewards that are associated with some delay. Such future rewards are typically devalued (discounted) over time, a phenomenon referred to as temporal (or delay) discounting [TD (2, 3)]. Although there is considerable variability in TD in healthy humans (4), addiction in particular is reliably associated with steep reward discounting (2). Patients suffering from substance-based addictions such as opioid (5, 6), cocaine (7), and nicotine addiction (8) as well as those suffering from non-substance-based addictions such as pathological gambling (PG) show increased TD (9–12). Although steep TD is consistently observed in gambling addiction (10), the underlying cognitive mechanisms are still unclear. For example, impairments in reward valuation processes and a lack of executive control might both contribute to steep discounting.

When faced with a decision between rewards, agents assign a subjective value to each option and compare options by comparing those values (13). An impaired valuation process in PG could be one possible contributing factor for impulsive decisions and steep TD in PG. Valuation processes are reflected in neuronal valuation signals in the brain’s rewards circuits (14). It is an ongoing debate whether value signals in, for example, the ventral striatum are decreased or increased in PG (15). While the reward deficiency hypothesis states that addiction is associated with diminished striatal value signals (16), an idea supported by some studies (17–19), other studies have found the opposite effect (20, 21). PG has been associated with higher levels of brain-derived neurotrophic factor (BDNF), which regulates midbrain dopamine release (22, 23). This effect was not associated with addiction severity (22). Monetary rewards might affect the reward system of PGs differently depending on the context and that the relation to non-monetary rewards is important. Given these mixed results, it cannot be ruled out that steep TD in PG is caused by disturbances in valuation processes.

Executive control is thought to be crucial to resist tempting immediate rewards. Thus, in addiction to deficits in reward valuation, executive control impairments might be an additional explanation for steep TD in PG. There is evidence for impairments of executive control in PG in a Stroop task and changes of the related neuronal activity in the left ventro-medial prefrontal cortex were reported (24). PG shares increased TD impulsivity with substance-use disorders [SUDs (25, 26)], but executive functions in general seem to be less impaired in comparison to SUDs. Nevertheless, these findings provide evidence that executive control in PG might be impaired in comparison to healthy control (HC) participants.

It has been speculated earlier (27) that one process that might support future-oriented decision-making in humans is episodic future thinking [EFT (28–30), sometimes also referred to as prospection]. The concept of EFT is closely linked to episodic memory. Episodic memory caches personal experiences and allows for “mental time travel” into the past (30–32). Episodic memory and personal semantic memory together constitute autobiographical memory [AM (1)]. A standard procedure to measure AM is the Autobiographical Memory Interview [AMI (1)], which assesses memories of personal events and differentiates between episodic memory and semantic information. In recent studies, variations of the AMI have been used to asses EFT as well [e.g., in Ref. (33)]. Recent cognitive neuroscience findings suggest that the same neuro-cognitive system consisting of medial temporal, parietal, and medial prefrontal networks supports projections of the self into the past and into the future (28–30, 32, 34–36). In accordance with this observation, AM and EFT are typically jointly impaired in many neurological and psychiatric disorders, e.g., in mild cognitive impairment (37), Alzheimer’s disease (38), hippocampal amnesia (39), and medial temporal lobe damage (33), as well as in, e.g., autism (40), post-traumatic stress disorder (41), and schizophrenia (42). However, given that steep discounting is critical to addiction psychopathology (for example, it is also associated with clinical relevant parameters such as relapse (43–45), it is surprising that relatively little is known about potential AM and EFT impairments in addiction in general and PG in particular. A recent study by Mercuri et al. (46) found impairments in EFT, but not in AM in a group of opioid addicts. Alcohol and opioid addicts use shorter time horizons in an imagination task (47, 48), but it is unclear if steep reward discounting in addiction is associated with impairments in EFT.

There is growing evidence that TD depends on and interacts with EFT. EFT, as measured by episodic details generated in the AMI, predicts TD rates in healthy adolescents (49). Moreover, it has been shown that TD and EFT interact both in terms of behavior and neuronal systems. Cueing EFT during a TD task led to less impulsive decisions and this change in behavior was associated with coupling of neuronal signals in the anterior cingulate cortex and the hippocampus (50). Likewise, participants are more willing to wait for rewards after vividly imagining the consumption of a delayed reward (51), an effect mediated by the medial rostral prefrontal cortex. However, also unspecific and relatively general imaginations about the future can lead to attenuated TD behavior (52). Additionally, a recent study reported that patients suffering from hippocampal damage do not show attenuate discounting when cued with EFT information, unlike HC participants (53). By contrast, TD behavior itself seems to be relatively unaffected by episodic amnesia (54, 55), suggesting that EFT might modulate TD, but is not a necessary process for TD. It has been suggested that EFT might affect TD not by modulation option values but by shortening the time horizon in general (56). This might explain why even relatively unspecific EFT interventions can influence TD choice behavior (52, 56).

It has been proposed that time perception is non-linear, which might contribute to the hyperbolic (rather than linear or exponential) shape of the discounting function, such that discounting is steeper over near vs. far time intervals (57–60). There is some evidence for impairments in time perception in the range of seconds to minutes in addicts (61). However, whether this extends to longer time spans relevant to discounting tasks (e.g., days to months) remains unclear. An altered subjective perception of time delays might at least partly explain the steeper TD in PG. Thus, a reliable assessment of time perception is important. We developed a novel task to assess the time perception of PGs in comparison to HC participants.

Given the separate evidence for interactions of TD with EFT and for steeper TD in addiction, it has been hypothesized that steeper TD, for example in gambling addiction, might, at least partly, be explained by EFT deficits (10). In the present study, we directly explore such a potential association. EFT was assessed using a modified version of the AMI (1), and TD was assessed using standard decision tasks. Additionally, we hypothesize that an altered subjective perception of time delays might contribute to steeper TD in addiction (62). Here, we examine time perception in gambling addicts and healthy participants using a novel task. We examined a group of gambling addicts and a group of HC participants matched for age, education, and smoking status.

## Materials and Methods

### Participants

We investigated 20 participants fulfilling the DSM-5 criteria of pathological gambling [age mean (range) = 32.9 (19–59), 19 males] and 20 healthy control participants [age mean (range) = 32.55 (18–58), 19 males]. Both groups were matched for age, education (completed school years), and smoking behavior assessed with the Fagerstrom test for nicotine dependence [FTND (63), see Table 1 for a sample overview]. We recruited all participants via advertisements on local internet bulletin boards. Participants received 10 EUR per hour as a compensation for participation. All participants provided written informed consent to participate. The local Institutional review board (Hamburg Board of Physicians) approved all study procedures.

**Table 1:** **Overview about tested sample and matching statistics**.

	HC	PG	Group comparison
Age median (range)	32.55 (18–58)	32.90 (19–59)	*t*_37.88_ = 0.09, *p* = 0.93
Education in school years median (range)	10 (9–13)	10 (9–13)	*t*_38_ = 0.74, *p* = 0.47
Monthly income in EUR mean (SD)	1042.90 (452.54)	1279.70 (1070.48)	*t*_25.58_ = 0.91, *p* = 0.37
FTND mean (SD)	3.5 (2.59)	4.45 (2.76)	*t*_37.84_ = 1.12, *p* = 0.27
AUDIT mean (SD)	7.15 (7.37)	10.3 (6.28)	*t*_37.07_ = 1.45, *p* = 0.15
DSM-IV pathological gambling mean (SD)	0.1 (0.31)	5.75 (1.74)	*t*_20.18_ = 14.27, *p* < 0.001
KFG mean (SD)	1.75 (2.71)	30.15 (7.74)	*t*_23.60_ = 15.48, *p* < 0.001
SOGS mean (SD)	0.45 (0.60)	9.65 (3.01)	*t*_20.53_ = 13.39, *p* < 0.001
BDI mean (SD)	4.6 (4.07)	16.5 (9.48)	*t*_25.77_ = 5.16, *p* < 0.001

*HC, Healthy control participants; PG, Pathological gamblers; SD, standard deviation; FTND, Fagerstrom Test for Nicotine Dependence; AUDIT, Alcohol Use Disorders Identification Test; KFG, Kurzfragebogen zum Glücksspielverhalten (a measure of gambling addiction); SOGS, South Oaks Gambling Screen; BDI, Beck Depression Inventory (see Materials and Methods for details)*.

### General procedure

Testing of EFT might induce a future-oriented mindset, which could affect TD (56). To avoid such carry-over effects, TD was always tested first, followed by the time perception and circle-size rating procedure. Then, EFT and AM were assessed before finally all questionnaire-based measures were completed. Testing took about 3 h in total.

### Psychological assessment

Severity of gambling addiction was assessed by the “Kurzfragebogen zum Glücksspielverhalten” [KFG (64)] and a German adaptation of the south oaks gambling screen [SOGS (65)]. Depression is a common co-morbidity in pathological gambling (66) and was assessed using the Beck Depression Inventory (67). Nicotine addiction was measured with the FTND (63), and alcohol consumption was measured with the alcohol use disorders identification test [AUDIT (68)].

### Autobiographical memory interview for past and future events

We assessed AM and EFT using a procedure similar to a study by Race and colleagues (33), based on the AMI (1). For EFT, participants were asked to imagine five events that could happen one year from now. To ensure comparability across participants, five short, unspecific cues were given (e.g., “Imagine someone special is visiting you one year from now. Describe in as much detail as you can what this event will be like. Describe where and when this event will be, who is there, how you feel, and what you are thinking”). Following each cue, the participant was asked to verbalize cue-related imagination for a maximum of 3 min. As soon as the participant finished, a standardized follow-up question was asked: “Can you tell me any more about where and when the event is taking place, who is there, how you feel, and what you are thinking?” For AM, participants were asked to recall and elaborate on five autobiographical episodes that happened a year ago. Again, to ensure comparability across participants, these episodes were cued (e.g., “Imagine a trip one year ago. Describe in as much detail as you can what this event was like. Describe where and when this event happened, who was there, how you felt, and what you were thinking.”) Again, elaborations were limited to 3 min. AM reports were followed by the same follow-up question as in the EFT condition.

All reports were recorded and transcribed. Due to technical reasons, only a subset of two (instead of five) AM events was recorded for one PG participant. All other participants completed all measures. In line with recent studies that applied the AMI (33, 38, 39, 69–72), reports were subsequently scored by one rater according to the AMI manual (1) to disentangle episodic from semantic content. Each informational detail was classified into one of several categories. The scoring manual differentiates sub-categories of episodic details: “Internal Event Details,” “Internal Place Details,” “Internal Time Details,” “Internal Perceptual Details,” and “Internal Emotion/Thought Details,” as well as semantic details (semantic information about the event) and external details. Details were rated as external if they fulfill the criteria of episodic details but belong to events other than the tested one (1). To assess the reliability of the rating procedure, two more raters scored a subset of 60 events. The overall inter-rater reliability was high (Cronbach’s α = 0.94).

### Time perception

Time perception was assessed using a novel task. Based on the assumption that time perception might be non-linear (57, 58, 73–75), we developed a task without an obvious linear structure (e.g., a bounded linear pen and paper scale might allow participants to be consistent across items, as a linear scaling of time perception could be mimicked by linear scaled answers). This might lead to more linear ratings because of the item arrangement and would be unrelated to delay perception. We therefore measured time perception via a manual adjustment of circle sizes. The procedure included two tasks: a time perception task to assess subjective time perception and a circle-size rating task to control for potential non-linearities (or individual differences) in circle time perception.

#### Time Perception Task

**Figure 1:**
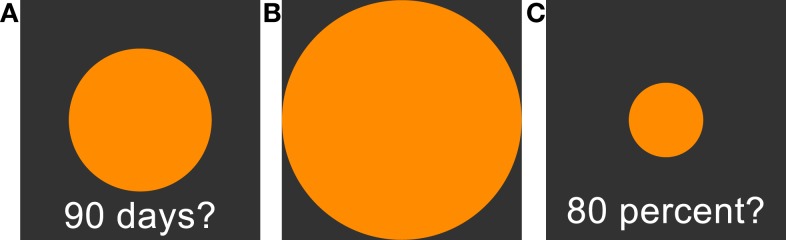
(**A**): Time perception task. Participants are asked to adjust the circle size in accordance to their estimation of subjective waiting time. Larger circles are indicating longer subjective waiting time. (**B**): 100% reference circle presented before the circle-size estimation task. (**C**): Circle-size estimation task. Participants are asked to adjust circle sizes in reference to the presented 100% reference circle (**B**). See Section “Materials and Methods” for further details.

All tasks were implemented in the Presentation software package (Neurobehavioral Systems, Inc.) on a standard personal computer. On a square subsection of the computer screen, a delay was displayed in the lower part as a text (see Figure 1). Participants were then asked to adjust the size (via arrow buttons) of a centrally presented circle according to their subjective perception of the respective duration of the delay. Pressing the right button enlarged the circle, pressing the left button shrank the circle. Circle size could expand beyond the screen, but scaling stopped shortly before the circle filled the complete screen. If the delay was perceived as short, participants were asked to shrink the circle. If the delay was perceived as long, participants were asked to enlarge the circle.

We tested 18 delays, ranging from 1 day to 190 days. The set of tested delays was unknown to the participants in advance. Delays were split into two symmetric testing sets, starting either with the shortest or the longest delay, separated by a 20 s break. Note that ceiling effects might induce non-linearity in the ratings. Ceiling effects could occur if short delay periods at the beginning of the testing session were rated as long (large circle sizes), leaving little space for longer delays. To avoid this, a rating using more than 90% of the scale led to longer delays being excluded from further testing. To avoid impulsive answers, proceeding to the next trial required a minimum of 10 s waiting period.

#### Circle-Size Estimation Task

Perception of circle sizes might in itself be non-linear and may confound the rating of delays. To address this issue, circle sizes were rated in a separate task. First, a circle touching all screen boundaries was shown to the participant and defined as a size of 100%. On each trial, participants were then asked to adjust a circle like the time perception task described in the previous section. However, instead of delays, target sizes in percent were shown, i.e., the circle had to be adjusted in size relative to the reference circle shown in the beginning. Participants had to rate all sizes of the time perception sessions, as well as standard reference sizes at 20, 40, 60, 80%. Again, the circle diameter was the dependent variable. Ratings of the circle-rating test were used to transform time perception ratings and to clean time perception ratings from influences of circle-size perception.

### Delay discounting

Delay discounting was tested with an adaptive task as described in previous studies (11, 76). Participants made repeated choices between a fixed smaller-but-sooner (SS) reward (20 EUR immediately) and varying larger-but-later (LL) rewards. Delays of LL rewards ranged from 1 day to 180 days in seven steps. Amounts of LL rewards were adjusted via a staircase procedure to converge at participant-specific indifference points for all seven delays (76). A second discounting task included trials by Kirby et al. (6, 77), consisting of 27 fixed items.

### Cognitive modeling

#### Time Perception – Model Selection and Parameter Estimation

**Figure 2:**
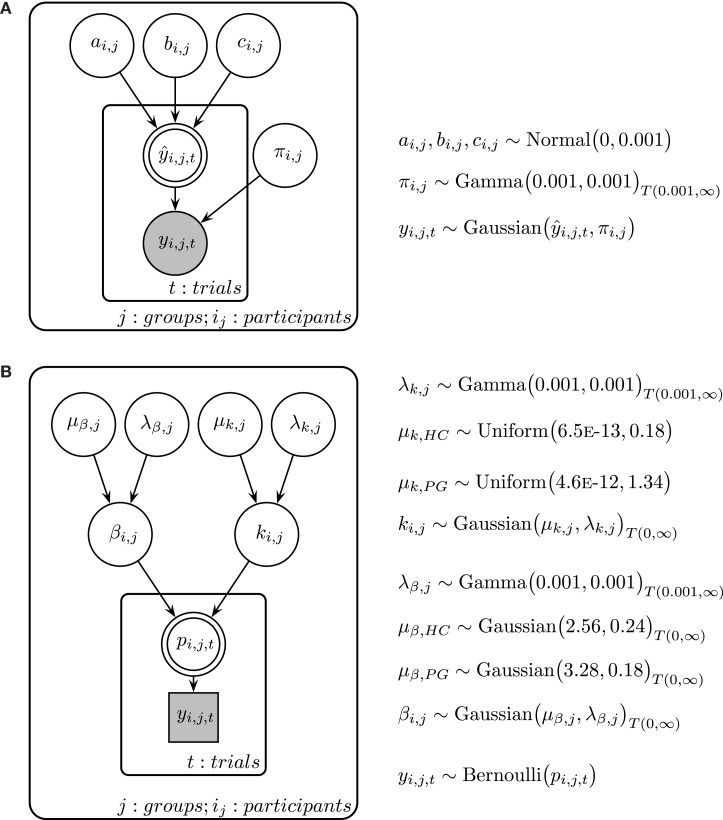
(**A**) Graphical description of the Bayesian model fit for time perception ratings and circle-size ratings. $\hat{y}$ is calculated with either a linear, a quadratic, or a power function, with the respective participant-specific parameters *a*, *b*, and *c* (see Materials and Methods in the text for more details). π is the participant-specific precision parameter and *y* is the measured ratings of the participant. (**B**) Graphical description of the Bayesian model fit for temporal discounting. μ_β_ is the group mean prior of the *β* parameter and λ_β_ is the respected precision parameter. μ*_k_* is the group mean prior of the *k* parameter and λ*_k_* is the respected precision parameter. Participant-specific *β* and *k* parameters are drawn from separate group distributions for HC and PG. *β* and *k* parameters determine the choice probability *p* in every trial, which is connected to the actual choice *y*. Shaded nodes indicate observed variables, while latent nodes are non-shaded. Round nodes are continuous quantities and squared nodes are discrete quantities. Double-bordered nodes are fully determined by their parent nodes.

Time perception ratings were analyzed individually for every participant. Adapting techniques from maximum likelihood estimation in linear regression, ratings for every delay were modeled using a linear function ($\hat{y}=\;a*x\;+\;b$), a quadratic function ($\hat{y}=\;a*x^{2}+\;b*x\;+\;c$), and a power function [$\hat{y}\;=\;a*x^{b}$, see also Ref. (57, 58)]. We assumed for every model that the observed ratings (*y*) are a combination of the true rating $\hat{y}$ and a constant error ϵ. Thus, we assumed the observed rating *y* to be Gaussian distributed with the true mean $\hat{y}$ and a participant-specific precision parameter π *(*i.e., inverse variance). All parameters were assumed to be constant within a participant. Posterior distributions for each participant were estimated with a Bayesian statistics approach, using Markov Chain Monte Carlo (MCMC) simulations in a Gibbs sampler [JAGS 3.3.0 (78)]. For more detail of the Bayesian model, see Figure 2A. All cognitive modeling of time perception was done in a single participant fashion to allow for a participant-specific assessment of goodness of model fit. Therefore, we calculated deviance information criteria [DIC (79)] for every model and every participant. DIC is a common criterion to estimate the goodness of model fit in Bayesian modeling, where smaller values indicate a better fit (79–81).

#### Circle-Rating – Model Selection and Parameter Estimation

Ratings of circle sizes were modeled with the same procedure as the time perception ratings using the same model equations. Again, posterior distributions of parameters were estimated using MCMC.

#### Temporal Discounting – Adaptive Testing

We analyzed participant’s choices in the TD tasks using a two-level cognitive model, where individual participant model parameters are assumed to be drawn from a common group-level distribution (see Figure 2B). In accordance with prior research (11, 82), we used a hyperbola to describe the decay of subjective value across time:

(1) $$\text{SV=}\frac{A}{1+k*D}$$

where SV is the subjective value of the presented larger-but-later (LL) reward, *A* is the amount of the LL reward in Euro, *k* is the participant-specific discounting parameter, and *D* is the delay of the LL reward in days. Second, the subjective value of the LL reward and the value of the 20 EUR immediate reward were passed to a softmax function to calculate choice probabilities for every trial:

(2) $$p_{\text{LL}}=\frac{\text{exp}\left({\text{SV}}_{\text{LL}}/\text{β}\right)}{\text{exp}\left({\text{SV}}_{\text{LL}}/\text{β}\right)+\text{exp}\left({\text{SV}}_{\text{SS}}/\text{β}\right)}$$

Here, *p*_LL_ is the probability to choose the LL option on a given trial, SV_LL_ and SV_SS_ are the subjective values of the LL and smaller-but-sooner options, and *β* is the subject-specific decision noise parameter. Participant-specific *k* and *β* parameters were estimated using a hierarchical Bayesian approach with separate group-level distributions for healty control participants and pathological gamblers (see Figure 2B for an overview of the estimation scheme). Hierarchical Bayesian estimation has been shown to result in more reliable parameter estimates compared to maximum likelihood approaches by sharing information among participants during the sampling process and providing measures of uncertainty for every parameter (83, 84). We used empirical prior distributions taken from previous studies of TD in pathological gamblers and HCs (11, 85).

#### Temporal Discounting – Task by Kirby et al.

TD choices of the Kirby et al. task (77) were analyzed with the same procedure as the choices of the adaptive TD task.

## Results

### Sample characteristics and psychopathology

We tested 20 participants fulfilling the DSM-5 criteria of pathological gambling [age mean (range) = 32.9 (19–59), 19 males] and 20 healthy control participants [age mean (range) = 32.55 (18–58), 19 males]. All particpants of the pathological gambling group differed significantly from HC in both gambling-related questionnaires, KFG and SOGS (see Table 1). Due to the positive correlation between both measures (*r* = 0.63, *p* = 0.003), we aggregated them into a single “addiction severity” score for all subsequent analyses by averaging *z*-transformed values across all participants. PGs also had higher levels of depression (BDI) compared to HCs (see Table 1). Both groups did not differ with respect to age (*t*_37.88_ = 0.09, *p* = 0.93), completed school years (*t*_38_ = 0.74, *p* = 0.47), monthly income (*t*_25.58_ = 0.91, *p* = 0.37), nicotine (*t*_37.84_ = 1.12, *p* = 0.27), and alcohol consumption (*t*_37.07_ = 1.45, *p* = 0.15).

### Episodic future thinking and autobiographical memory

**Figure 3:**
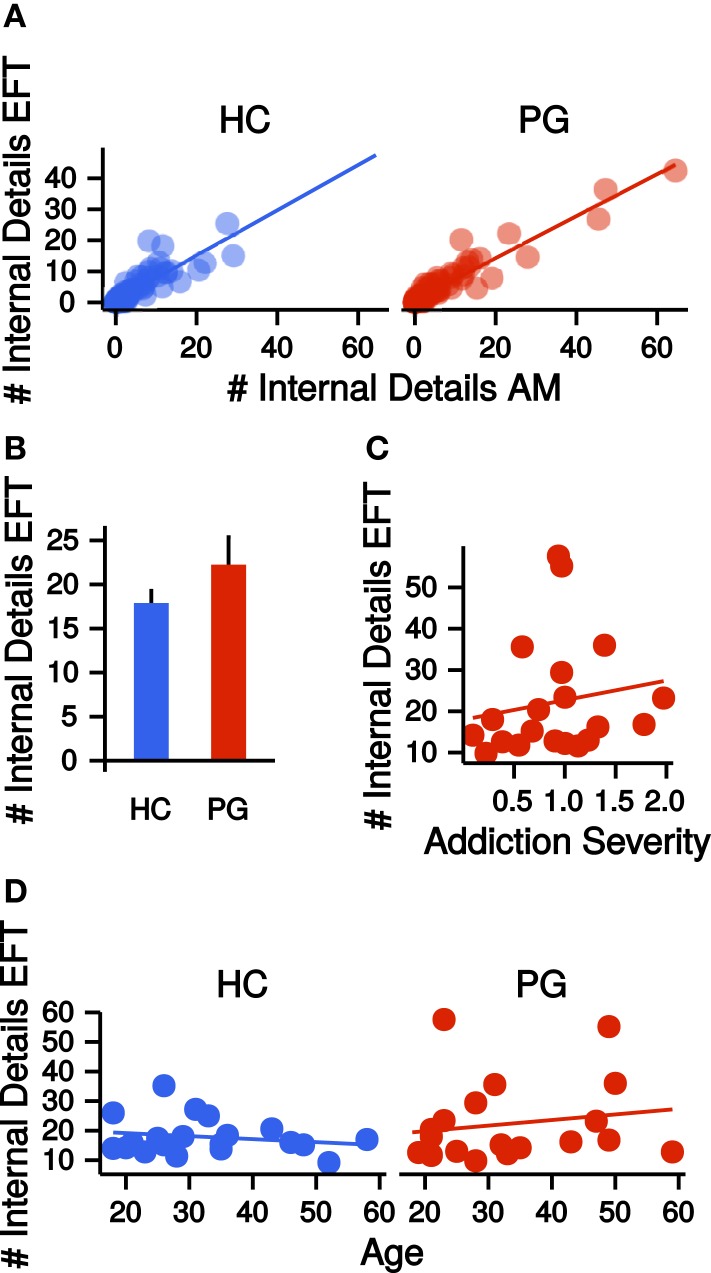
(**A**) Correlations of the number of internal details reported in the autobiographical memory (AM) condition with the number of internal details reported in the episodic future thinking (EFT) condition, separate for healthy control participants (HCs, *r* = 0.86, *p* < 0.001) and pathological gamblers (PGs, *r* = 0.95, *p* < 0.001). (**B**) Group difference in the number of internal details in the EFT condition. HC: healthy control participants, PG: pathological gamblers. Error bars are indicating SEM. (**C**) Correlation of number of internal details in the EFT condition with addiction severity scores in PGs (*r* = 0.16, *p* = 0.48). (**D**) Correlation of number of internal details in the EFT condition with age separate for HCs (*r* = −0.19, *p* = 0.42) and PGs (*r* = 0.16, *p* = 0.48).

Autobiographical memory and EFT were assessed via a modified version of the Autobiographical Memory Interview [AMI (1)]. Narratives were recorded, transcribed, and scored according to the AMI manual by Levine et al. (1) (see Materials and Methods). Similar to previous studies, we collapsed across all episodic detail sub-categories (“Internal Event Details,” “Internal Place Details,” “Internal Time Details,” “Internal Perceptual Details,” and “Internal Emotion/Thought Details”) per participant by first averaging detail subcategory scores across the five event cues and then summing all mean detail scores (internal details sum score). This was done separately for AM and EFT. Internal detail sum scores were highly correlated between AM and EFT (HC: *r* = 0.86, *p* = < 0.001; PG: *r* = 0.95, *p* = < 0.001, see Figure 3A). Groups did not differ on internal details sum scores (EFT: *t*_26.20_ = −1.27, *p* = 0.21, see Figure 3B, AM: *t*_26.71_ = −1.18, *p* = 0.25) and the correlation of these measures with addiction severity in PG was not significant (EFT: *r* = 0.16, *p* = 0.48, see Figure 3C, AM: *r* = −0.03, *p* = 0.91). There was no correlation between internal details sum scores and age, neither in HCs (EFT: *r* = −0.19, *p* = 0.42, AM: *r* = −0.00, *p* = 0.98) nor in PGs (EFT: *r* = 0.16, *p* = 0.48, AM: *r* = 0.16, *p* = 0.50, see Figure 3D). Completed school years correlated with EFT internal details in HCs (*r* = 0.46, *p* = 0.04), but not in PGs (*r* = −0.06, *p* = 0.80). Groups did not differ on semantic detail scores (EFT: *t*_30.91_ = −0.95, *p* = 0.35, AM: *t*_27.09_ = −1.21, *p* = 0.24) and the correlation of semantic details scores with addiction severity was not significant (EFT: *r* = 0.20, *p* = 0.40, AM: *r* = 0.11, *p* = 0.66). There was no correlation between semantic details scores and age in HCs (EFT: *r* = 0.35, *p* = 0.13, AM: *r* = 0.26, *p* = 0.28), but in PGs (EFT: *r* = 0.46, *p* = 0.04, AM: *r* = 0.40, *p* = 0.08). Semantic detail scores did not correlate with completed school years in both HCs (*r* = 0.15, *p* = 0.53) and PGs (*r* = −0.07, *p* = 0.77).

### Time perception

Time perception was assessed using a circle-size adjustment task. Participants were instructed to adjust circle sizes according to the perceived length of delays (see Materials and Methods). Time perception data were fit individually for each participant with a linear model, a power model, and a quadratic model using MCMC sampling (see Materials and Methods), yielding a DIC value per participant and model. Note that smaller DIC values indicate a better model fit. DIC values were summed across participants for each model separately. In both groups, a power law provided the best fit to the time perception rating data (see Table 2). Neither scaling parameter *a* or the curvature parameter *b* nor the precision parameter π differed between groups (*a*: *t*_35.50_ = −0.08, *p* = 0.94; *b*: *t*_35.48_ = 0.36, *p* = 0.72; π: *t*_37.58_ = −0.54, *p* = 0.59).

**Table 2:** **Model selection results: deviance information criterion (DIC) values of single participant model fits were summed across participants with respect to group**.

Analysis	Group	Model	Summed DIC
Delay rating	HC	linear	2422.38
	HC	quadratic	2365.70
	HC	power	**2303.22**
	PG	linear	2482.85
	PG	quadratic	2388.26
	PG	power	**2269.34**
Circle rating	HC	linear	2633.41
	HC	quadratic	2573.48
	HC	power	**2559.55**
	PG	linear	2568.90
	PG	quadratic	2484.61
	PG	power	**2463.32**
Corrected delay rating	HC	linear	2458.94
	HC	quadratic	2424.33
	HC	power	**2387.85**
	PG	linear	2401.46
	PG	quadratic	2348.61
	PG	power	**2251.79**

*Smaller values are indicating a better model fit. Bold typeface is indicating the smallest value per comparison*.

*HC, healthy control participants, PG, pathological gamblers*.

### Circle-size rating

To control for possible non-linear circle-size perception, an additional circle-size rating task was included. Again, goodness of fit for linear, quadratic, and power functions to the circle-size ratings was quantified via summed DIC scores across participants for each model. In both groups, a power law best accounted for circle-size perception (see Table 2). Scaling parameter *a*, curvature parameter *b*, and precision parameter π did not differ between groups (*a*: *t*_36.45_ = −0.71, *p* = 0.48; *b*: *t*_37.42_ = 0.48, *p* = 0.63, π: *t*_24.37_ = −1.27, *p* = 0.22).

### Time perception corrected by circle-size rating

The circle-size rating procedure revealed non-linear circle-size perception, that was, like the time perception data, best accounted for by a power law. To account for a possible influence of circle-size perception on time perception ratings, the time perception ratings were corrected by first inverting the power law of the circle-size rating. We transformed the time perception ratings by the participant-specific parameters of the circle-size estimation:

(3) $$\hat{y}={\left(\frac{y}{a}\right)}^{\frac{1}{b}}$$

where $\hat{y}$ is the corrected time perception rating, *y* is the original time perception rating, *a* is the participant-specific circle-size scaling parameter, and *b* is the participant-specific circle-size curvature parameter. Both *a* and *b* were obtained by fitting a power function to the results of the circle-size estimation procedure described above. Please note that data from participants that might have shown a linear circle rating will be transformed correctly (if *b* = 1, the power law becomes linear).

**Figure 4:**
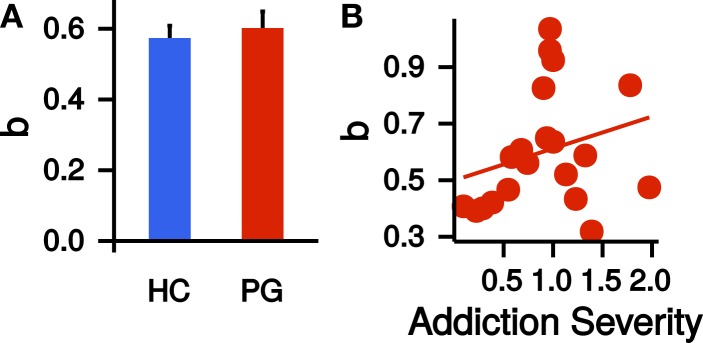
(**A**) Group difference in the exponent *b* of the power law time perception (corrected by circle-size estimation). HC, healthy control partici-pants; PG, pathological gamblers. Error bars are indicating SEM. (**B**) Correlation of exponent *b* of the power law time perception (corrected by circle-size estimation) with addiction severity scores in PGs (*r* = 0.26, *p* = 0.26).

We repeated the same model selection procedure (linear, quadratic, power) with the corrected time perception ratings. Note that these final ratings are corrected for potential non-linearities in circle-size perception. Again, a power model described the (corrected) time perception best (see Table 2). Scaling parameters *a*, curvature parameters *b*, and precision parameter π of the corrected time perceptions did not differ significantly between HCs and PGs (*a*: *t*_37.97_ = −0.16, *p* = 0.88; *b*: *t*_35.14_ = −0.48, *p* = 0.63; π: *t*_28.60_ = −1.76, *p* = 0.09; see Figure 4A). Within the PG group, neither *a* nor *b* correlated significantly with the addiction severity compound score (*a*: *r* = −0.03, *p* = 0.90, *b*: *r* = 0.26, *p* = 0.26, see Figure 4B).

### Temporal discounting

A hierarchical estimation of single participant *k* parameters was performed separately for the adaptive TD task and the Kirby task. The MCMC sampling estimates posterior distributions for every parameter and we used the median of the posterior as a summary measure for each participant’s posterior distribution. Because the distribution of *k* parameters in the sample was skewed, we applied a log-transformation to all *k* parameters. *k* parameters were highly correlated between the two discounting tasks in both groups (HC: *r* = 0.80, *p* < 0.001; PG: *r* = 0.87, *p* < 0.001). Therefore, we re-fited the combined datasets of both tasks for the remaining analyses. Again, we used the median as a summary measure of the posterior distribution and log-transformed all parameters to account for skewed parameter distributions.

**Figure 5:**
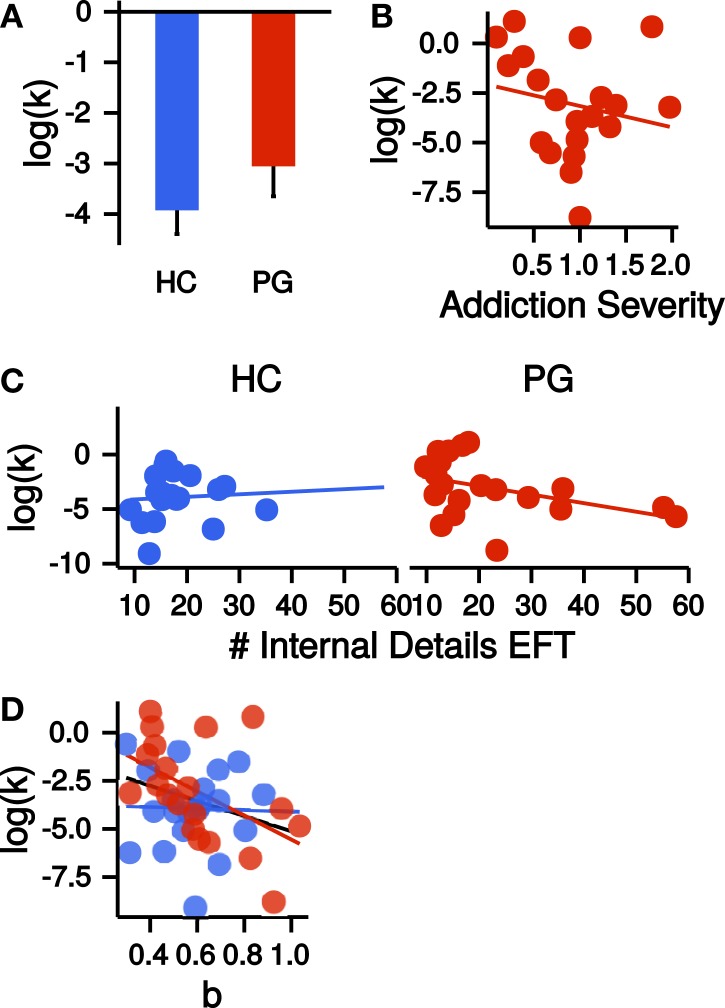
(**A**) Group difference in the log-transformed *k* parameter of the temporal discounting task. HC, healthy control participants, PG, pathological gamblers. Error bars are indicating SEM. (**B**) Correlation of log-transformed temporal discounting *k* parameters with addiction severity scores in PGs (*r* = −0.20, *p* = 0.40). (**C**) Correlation of the number of internal details in the episodic future thinking (EFT) condition with log-transformed temporal discounting parameters *k* separate for HCs (*r* = 0.07, *p* = 0.77) and PGs (*r* = −0.41, *p* = 0.07). (**D**) Correlation of exponent *b* of the power law time perception (corrected by circle-size estimation) with log-transformed temporal discounting parameters *k* across both groups (black, *r* = −0.30, *p* = 0.06) and separate for HCs (blue, *r* = −0.03, *p* = 0.90) and PGs (red, *r* = −0.50, *p* = 0.03).

Although *k* parameters were numerically greater in the PG group (see Figure 5A), the direct comparison was not significant (*t*_36.09_ = −1.15, *p* = 0.26, Cohen’s *d* = −0.36, see Figure 5A). Addiction severity did not correlate with *k* in PGs (*r* = −0.20, *p* = 0.40, see Figure 5B), which is consistent with prior research (10). *β* parameters, indicating decision noise, were not different between PGs and HCs (*t*_37.52_ = −0.66, *p* = 0.51). EFT internal details sum scores showed a non-significant trend-level correlation with *k* parameters in the PG group (*r* = −0.41, *p* = 0.07). Thus, in the PG group, better EFT ability tended to be associated with less discounting. This correlation was not present in the control group (*r* = 0.07, *p* = 0.77, see Figure 5C), but a direct comparison of the two correlations revealed no significant difference (*z* = 1.57, *p* = 0.12). A stronger curvature of time perception showed a trend-level association with steeper TD across groups (*r* = −0.30, *p* = 0.06). However, examination of this correlation separately for each group revealed that it was driven by a stronger correlation in PGs (*r* = −0.50, *p* = 0.03) in comparison to HCs (*r* = −0.03, *p* = 0.90, see Figure 5D). However, the correlations did not differ significantly between groups (*z* = 1.5, *p* = 0.13).

**Figure 6:**
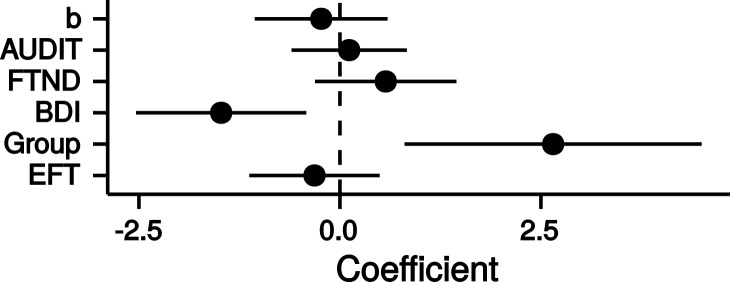
**Point estimates and 95% confidence intervals of variables predicting log(*k*) temporal discounting parameters**. *b*: exponent of the time perception function, *AUDIT*: Alcohol Use Disorders Identification Test (68), *FTND*: Fagerström Test for Nicotine Dependence (63), *BDI*: Beck Depression Inventory (67), *Group*: healthy control participants vs. pathological gamblers, *EFT*: mean number of internal details in episodic future thinking. All variables were *z*-transformed before entering the regression analysis.

Finally, we combined all measures [FTND, AUDIT, BDI, group (HC/PG) and time perception curvature parameter *b*] in a single multiple regression model with the dependent variable log(*k)*. Only the parameters for the predictors BDI and group were significantly different from zero (BDI: *b* = −1.48, *p* = 0.01; group: *b* = 2.65, *p* = 0.01; adjusted *R*^2^ = 0.20, see Figure 6). Thus, PG was associated with steeper discounting, whereas *higher* BDI was associated with reduced discounting.

## Discussion

We explored associations between temporal discounting, episodic future thinking [as measured using the Autobiographical Memory Interview (1)] and time perception in pathological gamblers and matched healthy control participants. Based on recent findings of an association between prospection and discounting (49–52), we speculated that impulsive choice in PG (i.e., steep reward discounting) might in part be attributable to attenuated prospection abilities. However, groups did not differ in baseline prospection (i.e., episodic future thinking internal details scores). In both groups, time perception was best accounted by a power law, and the degree of non-linearity in time perception correlated with temporal discounting across groups. A final multiple regression analysis across all predictors revealed that only group status (PG/healthy control participants) and depression, but not episodic future thinking or autobiographical memory, were significantly associated with discounting behavior.

In line with the conceptual framework of constructive episodic simulation (29, 30) and consistent with previous findings (29, 35), episodic future thinking (EFT) was highly correlated with autobiographical memory (AM) in pathological gamblers (PGs) and healthy control participants (HCs) alike. Neither EFT nor AM differed between groups, and neither correlated with addiction severity in the PG group. Psychiatric disorders such as autism (40), post-traumatic stress disorder (41), and schizophrenia (42) have been associated with changes in EFT. We hypothesized that impulsive choice behavior in addiction and PG could be in part explained by less detailed imaginations of the future. The present study suggests that PG may not be associated with similar impairments. Of note, PG was taken as a model for human addiction that is not confounded by effects of neuro-toxic substances (25, 86). However, it is possible that EFT and/or AM deficits might be more pronounced in SUDs. A recent study by Mercuri et al. (46) found EFT, but not AM, impairments in long-term opiate users. In alcohol abuse, neuro-toxic effects of heavy alcohol consumption can lead to Korsakoff’s syndrome, which includes memory impairments (87). These memory impairments might impact EFT as well.

Across groups, more vivid EFT was not associated with reduced TD. However, a greater number of EFT details tended to be associated with less impulsive TD in PGs. In a previous study using a very similar approach, individual differences in EFT predicted discounting behavior in a sample of healthy adolescents after controlling for questionnaire-based impulsivity, intelligence, development, autobiographic memory, and semantic future details (49). This finding suggests a specific impact of future event imaginations on future decision-making in healthy adolescents. Although the regression coefficient for EFT was not significantly different from zero, it was overall negative as in the previous study (49). This previous finding provides evidence for a relation of vivid EFT and shallow TD, which could not be replicated in the present sample of healthy adults. Future studies with larger samples are required to explore how AMI-based prospection measures relate to discounting behavior in healthy adults. Furthermore, it remains unclear why the association between discounting and EFT appeared to be more pronounced in PGs than HCs. Prior research found evidence for subtypes of PG (88, 89), including a subtype summarized as “antisocial impulsivist” [e.g., Ref. (90–92)]. Although the present sample size precludes us from investigating PG subtypes, one could speculate that this subtype that is associated with feelings of enhancement, could lead to more extensive narrations, which might mask other effects in the present study.

Episodic future thinking depends on a brain network consisting of the medial temporal lobe, the medial prefrontal cortex, and the retrosplenial cortex/posterior cingulate (34–36). Valuation signals of immediate and delayed rewards have been observed in the ventral striatum and ventro-medial prefrontal cortex (76), while the specific changes of valuation signals in the ventral striatum in PG are still debated (16–19). Growing evidence supports the idea that vivid EFT can attenuate TD under some conditions (50–52, 93). Prefrontal–medio-temporal interactions have been identified to support these changes in TD behavior (50, 51). The present results of our study are not in line with the hypothesis that steep TD in PG might be due to attenuated EFT. Moreover, if anything, PGs showed numerically greater internal details scores than controls.

Since the primary aim of the present study was an assessment of baseline EFT capabilities in PG, EFT and TD were tested in two separate tasks. However, the lack of overall group differences in EFT does not rule out the possibility that EFT–TD interactions might be diminished in PG under different task conditions. For example, it is unclear if PGs show effects of episodic tagging (cueing) during discounting, which have been repeatedly shown in HCs (50–52, 56, 93). Such beneficial effects of EFT on TD would be of interest for the development of novel therapeutic approaches for PG. Since the present results suggest that EFT is not generally impaired in PG, this might be an interesting avenue for future research.

We replicated previous findings of non-linear time perception [power law (57, 58)] in both PGs and HCs. We applied a novel task, utilizing a non-linear rating procedure via circle-size adjustments. This enabled us to disentangle general circle-size perception from delay perception. Scaling and curvature parameters of the power law describing delay perception did not differ between groups and were not correlated with addiction severity in the PGs. The finding of non-linear time perception in both groups suggests that TD differences between HCs and PGs are unlikely to be attributable to differences in delay perception. By contrast, time perception deficits have been reported for SUD when estimating shorter time spans [<1 min (61)]. Additionally, we explored whether steep TD in PGs might be (in part) related to shortened time horizons. In line with prior research, attenuated non-linear time perception was associated with steeper discounting across both groups (57, 58). Although this effect appeared to be driven by a stronger effect in the PG group, the correlations did not differ significantly. Steeper TD in PG can therefore also not be explained by shortened time perception. It has been argued that interventions that use EFT to reduce TD might lengthen participants’ subjective time horizons (such that options with longer delays are taken more into account) rather than shifting valence or attentiveness of the delayed rewards (56). Although this might be relevant to studies examining effects of EFT directly during decision tasks [e.g., Ref. (50)], it is not directly relevant to our study, as we assessed TD and EFT separately.

Temporal discounting behavior was assessed using two tasks: first, an adaptive task using a staircase procedure was applied (76). Second, a standard task by Kirby et al. (6) using 27 fixed trials was performed. *k* parameters estimated from both tasks were highly correlated, reflecting the high reliability of *k* parameters. After controlling for smoking behavior (FTND), alcohol use (AUDIT), non-linearity of time perception (exponent *b*), and EFT episodic details, only depression (as measured by the BDI) and group status (HC/PG) significantly predicted TD. The group difference was absent in a direct comparison of *k* parameters between groups in a *t*-test, likely due to masking effects of the covariates, which are removed in the regression analysis. Higher BDI scores were associated with lower *k* parameters while PG group status was associated with higher *k* parameters. Similar to PG, which is consistently associated with impulsive TD (9–12), major depressive disorder and bipolar disorder both have been associated with steeper discounting (94–97) and it has been speculated that this might reflect a state of hopelessness (94, 95). Depression is a common co-morbidity in PG (98) and might differentially affect reward processing in PG. A recent study by Fauth-Bühler et al. (99) found a positive interaction of PG and depression on reward signals in the right insular cortex and in the ventral striatum during an effort-dependent monetary reward task. This finding indicates a possible interaction of depression and PG in reward processing on a neuronal level. However, these interactions might not always manifest in behavioral differences (99). In our study, only PG and depression significantly influenced discounting behavior. However, the directions of effect were opposed, as PG intensified TD and depression attenuated TD.

Specific TD testing conditions might have affected our results. All smaller-but-sooner options were immediate offers, which might lead to more impulsive choices [a phenomenon known as the immediacy effect (100)]. All TD rewards in our study were hypothetical. It has been shown that real rewards lead to attenuated discounting behavior (101–103). How this effect might interact with PG is unclear, but earlier studies found group differences between HC and PG using (potentially) real rewards (11, 104). Additionally, the role of reward magnitudes remains to be explored, both with respect to effects in PG and with respect to EFT modulations. It is possible that experiments using larger reward magnitudes (>1000 EUR) might be more sensitive to detect correlations with EFT and/or time perception.

Taken together, EFT and AM were highly correlated in both healthy control participants and pathological gamblers, and neither process was impaired in the pathological gamblers. Likewise, we found no evidence for changes in anticipatory delay perception in pathological gambling. These findings suggest that steep discounting in pathological gambling is unlikely to be due to impairments in general prospection abilities or due to skewed representations of time delays. If anything, gamblers produced more future event details than controls. Interactions between EFT and TD remain an important issue for future research in addiction neuroscience and psychiatry. Further studies are required to assess whether the present findings extend to substance-use disorders and other disorders of impulse control that are characterized by impulsive and short-sighted behavior.

## Author Contributions

AW, UB, and JP conceived and designed the study. AW acquired the data. AW and UB analyzed the data. AW and JP wrote the manuscript.

## Conflict of Interest Statement

The authors declare that the research was conducted in the absence of any commercial or financial relationships that could be construed as a potential conflict of interest.
